# Insampaedok-San Extract Exerts an Immune-Enhancing Effect through NF-*κ*B p65 Pathway Activation

**DOI:** 10.1155/2023/5458504

**Published:** 2023-09-22

**Authors:** Gyuwon Huh, Youngse Oh, Youngsic Jeon, Ki Sung Kang, Su Nam Kim, Sang Hoon Jung, Seung Hyun Kim, Young-Joo Kim

**Affiliations:** ^1^Natural Product Research Center, Korea Institute of Science and Technology, Gangneung 25451, Republic of Korea; ^2^Division of Bio-Medical Science & Technology, University of Science and Technology, Daejeon 34113, Republic of Korea; ^3^College of Pharmacy, Yonsei Institute of Pharmaceutical Science, Yonsei University, Incheon 21983, Republic of Korea; ^4^College of Korean Medicine, Gachon University, Seongnam 13120, Republic of Korea

## Abstract

Insampaedok-san (IS) has traditionally been prescribed as a medication for cold-related symptoms in Northeast Asia, including Korea and China. In this study, we focused on elucidating the molecular mechanism underlying the immunomodulatory activity of IS water extract (ISE) in macrophages. ISE significantly enhanced the levels of nitric oxide (NO) and prostaglandin E2 (PGE_2_) by increasing the expression of inducible NO synthase and cyclooxygenase-2 (COX-2) in a dose-dependent manner. ISE, which consists of many herbs, contains a large number of active compounds whose pharmacological targets and mechanisms are complicated. Therefore, network pharmacology analysis was used to predict the potential key components, targets, and mechanisms of ISE as immunomodulators. Subsequently, the network pharmacology results were validated experimentally. Seven key components were identified through HPLC-QTOF-MS. As predicted by the network pharmacology analysis, ISE increased the mRNA expression of *Tnf* and *Il6*. Furthermore, ISE increased the phosphorylation, nuclear translocation, and transcriptional activity of the p65 subunit of the nuclear factor-*κ*B (NF-*κ*B) signaling pathway. In contrast, rapamycin, an NF-*κ*B inhibitor, suppressed the ISE-induced mRNA expression of *Tnf* and *Il6*. In conclusion, ISE is an immune activator that can elevate the production of NO, PGE_2_, and proinflammatory cytokines mediated by NF-*κ*B signaling.

## 1. Introduction

The COVID-19 pandemic has aroused global attention regarding the importance of preexisting immunity. Recent data have shown that enhanced immune responses derived from prior infection with the common cold may protect against COVID-19 [1]. Immunity, a biological defense response against pathological threats including invasion of pathogens or disturbed homeostasis of the human body, can be categorized into innate immune responses associated with macrophages and natural killer cells, as well as adaptive immune responses related to T and B lymphocytes [2].

Phagocytes, such as neutrophils, monocytes, and macrophages, are the primary cells involved in the nonspecific immune response that defends the human body in the event of microbial invasion [3]. Among phagocytes, macrophage activation is indispensable for the progression of inflammatory diseases through the release of cytokines such as interleukin-6 (IL-6) and tumor necrosis factor-*α* (TNF-*α*), as well as other inflammatory regulators such as nitric oxide (NO) and prostaglandin E2 (PGE_2_) [4, 5]. During the immune response, a large amount of NO is produced by inducible NO synthase (iNOS), and PGE_2_ is generated by the conversion of arachidonic acid catalyzed by cyclooxygenases (such as COX-1 and COX-2). Therefore, enhancement of NO and PGE_2_ production may be a valuable strategy for increasing immunity.

The nuclear factor-*κ*B (NF-*κ*B) signaling pathway plays an important role in regulating acute-phase immune responses. NF-*κ*B is activated by lipopolysaccharide (LPS) and proinflammatory cytokines, resulting in the phosphorylation and proteasomal degradation of I-*κ*B*α* (the inhibitor of *κ*B*α*) [6]. p65, a subunit of NF-*κ*B, can translocate from the cytosol to the nucleus, promoting the transcriptional activity of several target genes encoding immune regulators or cytokines, including iNOS, COX-2, TNF-*α*, and IL-6, by binding to *κ*B-binding sites [7, 8].

Insampaedok-san (IS, *Renshen bai du-san* in Chinese or *Ninjinhaidoku-san* in Japanese) has been widely prescribed as a treatment for cold-related symptoms in Korean and Chinese traditional medicines. In this study, we used a ten-herb formula that consists of *Glycyrrhiza uralensis* Rhizoma, *Ostericum koreanum* Radix and Rhizoma, *Platycodon grandiflorum* Radix, *Aralia continentalis* Radix, *Bupleurum falcatum* Radix, *Panax ginseng* Radix, *Angelica decursiva* Radix, *Poria cocos* Sclerotium, *Citrus aurantium* Fructus, and *Cnidium officinale* Rhizoma [9]. Considering the herbal composition of IS and its uses, it can be predicted that IS may modulate immune responses and affect the immune-related targets mentioned above. However, the diverse ingredients in IS, complex multitargets, and their interactions make it difficult to predict immune-related molecular mechanisms. These difficulties often occur in the study of a single herb containing many compounds and multiherb compositions. Recently, advances in network pharmacology analysis have attracted attention as a new approach for interpreting unclear molecular mechanisms of herbal medicines [10]. In this study, we applied this approach to more efficiently predict the key components, target proteins, and their related signaling pathways for the immune-enhancing effects of IS water extract (ISE). In addition, the network pharmacology results were experimentally validated in terms of both chemical and pharmacological aspects. Therefore, we determined the molecular mechanism underlying the immune response induced by ISE in RAW264.7 cells in this study.

## 2. Materials and Methods

### 2.1. Insampaedok-San Water Extract (ISE) Preparation

ISE was prepared from a chopped herbal mixture by Hanpoong Pharmaceutical Co., Ltd. (Jeonju, Korea) (Table 1). The mixture was added to 125 mL of distilled water and decocted at 90–100°C for 3 h. The extract was filtered through filter paper that had a 5-micrometer pore size, the filtrate was concentrated using an evaporator, and the remaining mass was vacuum-dried to obtain a powder. The ISE powder was dissolved in dimethyl sulfoxide (DMSO) for *in vitro* experiments.

### 2.2. High-Performance Liquid Chromatography Mass Spectrometry (HPLC-MS) Analysis

ISE powder (10.0 g) was dissolved in 100 mL of distilled water and subjected to ultrasonic extraction at room temperature for 60 min. The extract was filtered through filter paper (5–8 *μ*m, 150 mm) and concentrated under reduced pressure until dry. The concentrated sample was dissolved in HPLC-MS grade water (J.T.Baker®, Phillipsburg, USA) and filtered using a 0.2 *μ*m nylon syringe filter before HPLC-QTOF-MS analysis.

### 2.3. HPLC-QTOF-MS Analysis

HPLC was performed using an Agilent 1290 HPLC system (Agilent Technologies, Santa Clara, CA, USA) interfaced with an Agilent 6530 QTOF mass spectrometer (Agilent Technologies). An electrospray ionization (ESI) source was utilized in the positive ion mode to detect the key components. The parameters were as follows: mass range, 100–3200 m/z; nebulizer pressure, 35 PSIG; drying gas temperature, 300°C; drying gas flow, 8.0 L/min; capillary voltage, 3500 V; fragmentor, 175 V; skimmer, 65 V; and OCT 1 RF Vpp, 750 V. To obtain the exact mass of the key constituents of ISE, calibration was performed with an Agilent tune mix (Agilent Technologies) from 100 to 1600 Da. Separation was performed using a Waters® ACQUITY UPLC BEH C18 (2.1 mm i.d.×100 mm, 1.7 *μ*m). The mobile phase was composed of 0.1% formic acid in water as eluent A and 0.1% formic acid in acetonitrile as eluent B, with the following gradient elution profile: 5–95% B (0–20 min) and 100% B (20.1–25 min); equilibration was performed with 5% B for 3 min at a flow rate of 0.4 mL/min. The temperature of the column was maintained at 40°C. Each sample was injected in a volume of 5 *μ*L. All MS data were acquired using the MassHunter Qualitative Analysis software (version B.06.00; Agilent Technologies) to ensure mass accuracy throughout the chromatographic analysis. The “Find by Auto MS/MS” function of the analysis software was used to extract all the MS/MS spectra and identify compounds by comparing their accurate *m*/*z* values, isotope distributions, and fragment ions to the database.

### 2.4. Cell Culture

The mouse macrophage RAW264.7 cell line was purchased from the American Type Culture Collection (ATCC, VA, USA). The murine cells were cultured in Dulbecco's Modified Eagle's Medium (DMEM, Corning, VA, USA) supplemented with 10% fetal bovine serum (FBS, Gibco Ballistic Research Laboratory (BRL), MD, USA) and 1% penicillin and streptomycin (Gibco BRL, MD, USA). The cells were incubated at 37°C in a humidified atmosphere with 5% CO_2_.

### 2.5. Cell Viability

RAW264.7 cells were stimulated with 100–400 *μ*g/mL of ISE for 24 h, and cell proliferation was measured using a Quanti-Max™ WST-8 Cell Viability Assay Kit (Biomax QM3000, Seoul, Republic of Korea) according to the manufacturer's manual.

### 2.6. NO Assay

NO production was measured using the Griess assay, which measures the concentration of nitrite (NO_2_^−^) in the growth medium [11]. In brief, RAW264.7 cells were seeded in 96-well plates at a density of 1 × 10^5^ cells/well and incubated at 37°C in a humidified atmosphere with 5% CO_2_ for 6 h until they attached to the plates. The attached cells were treated with phenol red-free DMEM containing the indicated concentrations of ISE or 10 ng/mL of LPS (*Escherichia coli* strain 055:B5, Sigma-Aldrich, Missouri (MO), USA) for 24 h. The supernatant was transferred, mixed with an identical volume of the Griess reagent (1% sulfanilamide, 0.1% N-(1-naphthyl)-ethylenediamine, and 5% phosphoric acid), and incubated at room temperature for 5 min. The absorbance was determined using a microplate reader at a wavelength of 540 nm. Sodium nitrite was used in 2-fold and 7-point serial dilutions from 100 mM to create a standard reference curve. The control group of cells was treated with phenol red-free DMEM containing 0.5% (*v*/*v*) DMSO, and all *in vitro* experiments were performed in triplicate.

### 2.7. PGE_2_, TNF-*α*, and IL-6 ELISA

The murine macrophages were seeded in 96-well plates at a density of 1 × 10^5^ cells/well and incubated at 37°C in a humidified atmosphere with 5% CO_2_ for 6 h until they attached to the plates. The attached cells were exposed to DMEM without phenol red, which contained the indicated concentrations of ISE or 10 ng/mL LPS for 24 h. The supernatant was collected and stored at –80°C. PGE_2_ production was examined through the PGE_2_ Parameter™ Assay Kit (R&D Systems, Minnesota, USA). Moreover, the secretion of TNF-*α* and IL-6 was evaluated through the Ezway Cytokine ELISA Kit (LABISKOMA, Seoul, Republic of Korea). The levels of PGE_2_ and cytokines were determined using sandwich ELISA, according to the manufacturer's protocol.

### 2.8. Total RNA Extraction and Quantitative Real-Time PCR (qRT-PCR) Analysis

Cells were exposed to either LPS or ISE for 12 h; thereafter, total RNA was extracted using the RNeasy Mini Kit (Qiagen, MD, USA), and cDNA was synthesized using the RevertAid First Strand cDNA Synthesis Kit (Thermo Fisher Scientific, Vilnius, Lithuania) according to the manufacturer's protocol. cDNA samples (50 ng) were subjected to qRT-PCR analysis using a QuantStudio 6 Pro Real-Time PCR System (Applied Biosystems, Foster City, CA, USA), TaqMan® Gene Expression Assay (Applied Biosystems) (Table 2), and TaqMan® Fast Advanced Master Mix (Applied Biosystems). The cycling conditions were as follows: predenaturation at 95°C for 20 s, followed by 40 cycles of denaturation at 94°C for 1 s, annealing at 60°C for 20 s, and extension at 60°C for 20 s.

### 2.9. Fractionation of Cytosolic and Nuclear Extracts

RAW264.7 cells seeded in 100 mm dishes were treated with 10 ng/mL of LPS or with the indicated concentration of ISE for 1 h. The fractionation of cytosolic and nuclear extracts of RAW264.7 cells followed the method by Kim et al. [11]. Briefly, the stimulated cells were washed twice with cold phosphate-buffered saline (PBS). The cell pellets were lysed in hypotonic buffer (10 mM 4-(2-hydroxyethyl)piperazine-1-ethanesulfonic acid (HEPES, pH 7.9), 0.1 mM ethylenediaminetetraacetic acid (EDTA), 10 mM KCl, 1 mM dithiothreitol (DTT), 1 mM phenylmethylsulfonylfluoride (PMSF), 1× protease inhibitor cocktail, and 1 mM sodium orthovanadate (Na_3_VO_4_)) at 4°C for 15 min, and thereafter, 10% Nonidet P-40 (USB, OH, USA) was added. The mixture was vortexed and then centrifuged at 16,300 × *g* for 30 min at 4°C. The supernatant containing the cytosolic protein lysate was transferred and stored at –80°C. The nuclear pellets were washed twice with cold PBS and lysed in hypertonic buffer (20 mM HEPES (pH 7.9), 0.1 mM EDTA, 0.4 M NaCl, 1 mM DTT, 1 mM PMSF, 1× protease inhibitor cocktail, and 1 mM Na_3_VO_4_) for 15 min on ice. The lysed nuclear fraction was then centrifuged at 16,300 × *g* for 30 min at 4°C. The supernatant containing nuclear protein lysate was stored at –80°C.

### 2.10. Western Blot Analysis

RAW264.7 cells were grown in 6-well plates and treated with 10 ng/mL of LPS or an indicated concentration of ISE for 1 h or 24 h. Cells were then lysed using radioimmunoprecipitation assay (RIPA) buffer (Cell Signaling, MA, USA) supplemented with 1× protease inhibitor cocktail and 1× phosphatase inhibitor cocktail, according to the manufacturer's protocols. Proteins (whole-cell extracts, 30 *μ*g/lane; nuclear extracts, 10 *μ*g/lane; and cytosolic extracts, 10 *μ*g/lane) were isolated through sodium dodecyl sulfate polyacrylamide gel electrophoresis (SDS-PAGE) using Bolt™ 4-12% Bis-Tris Plus gels (Invitrogen, CA, USA) following the modified version of a previous protocol [12], transferred onto polyvinylidene difluoride (PVDF) membranes, and analyzed using epitope-recognizing primary and secondary monoclonal antibodies. Bound antibodies were visualized using SuperSignal™ West Femto Maximum Sensitivity Substrate (Thermo Scientific, IL, USA) and an LAS 4000 imaging system (Fujifilm, Japan). The monoclonal antibodies against iNOS, COX-2, phospho-NF-*κ*B/p65 (Ser536), NF-*κ*B/p65, HDAC1, phospho-ERK1/2 (Thr202/Tyr204), ERK1/2, phospho-p38 MAPK (Thr180/Tyr182), p38 MAPK, phospho-JNK (Thr183/Tyr185), JNK, and GAPDH were purchased from Cell Signaling Technology Inc. (Boston, MA, USA).

### 2.11. Dual-Luciferase Reporter Assay

RAW264.7 cells seeded in 96-well plates were cotransfected with pNF-*κ*B-Luc (BD Biosciences, CA, USA) and pNL-Luc (Promega, WI, USA) vectors using FuGENE 6 Transfection Reagent (Roche, Germany) according to the manufacturer's instructions. After cotransfection for 24 h, the cells were treated with the indicated concentration of ISE or 10 ng/mL of LPS for 6 h. Then, the cells were lysed and luciferase activity was measured using a dual-luciferase reporter assay system (Promega) and GloMax® Navigator Microplate Luminometer (Promega) according to the manufacturer's manual. The relative firefly luciferase activity was normalized to Renilla luciferase expression to adjust for variations in transfection efficiency. All *in vitro* experiments were performed in triplicate.

### 2.12. Network Pharmacological Analysis

The chemical components of ISE were manually collected from databases, including a database of medicinal materials and chemical compounds in Northeast Asian traditional medicine (TM-MC, https://informatics.kiom.re.kr/) [13] and PubMed (https://pubmed.ncbi.nlm.nih.gov/). All compounds were evaluated for their drug-likeness (DL) and oral bioavailability (OB) using the quantitative estimate of drug-likeness (QED) method. The physicochemical properties of the components were obtained using the SwissADME database (https://www.swissadme.ch/) [14] and input into the QED function to calculate the QED value of each compound [15]. The expected active compounds that satisfied the cut-off values (QED ≥ 0.4 and OB = TRUE) were selected. The SMILES strings of the expected active compounds of ISE and the SwissTargetPrediction database (http://www.swisstargetprediction.ch/) [16] were used to obtain the predicted targets. Immune-enhancing related targets were collected from the GeneCards database (https://www.genecards.org/) [17]. Venny 2.1.0 (http://bioinfogp.cnb.csic.es/tools/venny/) was used to draw a Venn diagram to detect common targets and filter potential targets which exhibit a relevance score ≥ 9. Specific protein class information of the potential targets was obtained from the DiGeNET database (https://www.disgenet.org/search) [18]. Protein-protein interactions were constructed to select the key targets from the potential targets based on three topological results (degree ≥ 10, betweenness centrality ≥ 0.001, and closeness centrality ≥ 0.6) [19] using the STRING database (https://string-db.org/) [20] and specific parameters (0.700, high confidence; 5%, medium FDR stringency). Pathway enrichment analysis was performed using the DAVID Bioinformatics Resources 6.8 database (https://david.ncifcrf.gov/home.jsp) [21]. The analysis parameter (*P* < 0.05) was set as the threshold value to identify significant signaling pathways. Gene Ontology (GO) and Kyoto Encyclopedia of Genes and Genomes (KEGG) pathway enrichment analysis results were visualized using ImageGP (http://www.ehbio.com/ImageGP). An integrated network of compounds, key targets, and pathways was established and analyzed using Cytoscape (v3.9.0; Seattle, WA, USA).

### 2.13. Statistical Analysis

Data are presented as the mean ± standard deviation (S.D.) of at least three independent readings for each experiment. Deviations between the means were evaluated by performing Student's *t*-tests.

## 3. Results

### 3.1. Effects of ISE on Cell Viability

**Figure 1:**
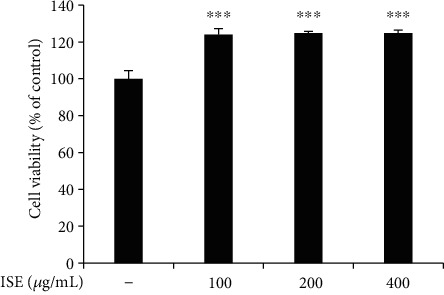
Effects of ISE on cell viability in RAW264.7 cells. Cells were exposed to growth medium in the absence or presence of ISE at 100–400 *μ*g/mL for 24 h. Cell viability was analyzed using the WST-8 assay. The data are shown as mean ± SEM of triplicate experiments. *P* values by unpaired Student's *t*-test. ^∗^*P* < 0.05, ^∗∗^*P* < 0.01, and ^∗∗∗^*P* < 0.001.

The cytotoxicity of ISE on RAW264.7 mouse macrophages was examined. ISE did not affect cell viability for 24 h at concentrations ≤ 400 *μ*g/mL (Figure 1). Based on this result, 100–400 *μ*g/mL ISE was used for subsequent experiments.

### 3.2. ISE Enhances NO and PGE_2_ Production

**Figure 2:**
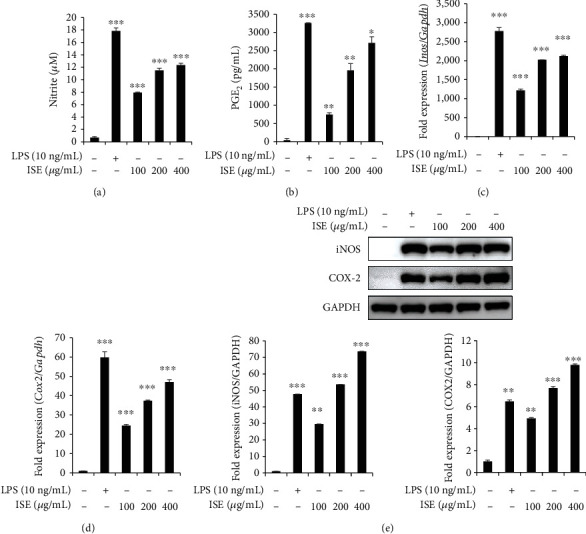
Effects of ISE on NO and PGE_2_ production in RAW264.7 cells. Cells were treated with phenol red-free medium (a, b) or growth medium (c–e) containing the indicated concentration of ISE or 10 ng/mL of LPS for 24 h. (a) The nitrite concentration was analyzed using the Griess assay. (b) The amount of PGE_2_ was measured using the PGE_2_ ELISA Kit. (c, d) mRNA expression of iNOS and COX-2 was analyzed using qRT-PCR. GAPDH was used as an internal control. (e) Protein expression of iNOS and COX-2 was measured through western blotting. GAPDH was used as an internal control. Bar plots show the densitometric analysis of (e). The data are shown as mean ± SEM of triplicate experiments.

NO and PGE_2_ are important proinflammatory mediators produced during inflammatory responses in macrophage cell lines [22, 23]. The immune-enhancing effects of ISE were evaluated using an NO assay and PGE_2_ ELISA, and it was found that ISE significantly enhanced NO and PGE_2_ production in a concentration-dependent manner compared with the control (Figures 2(a) and 2(b)). Additionally, we evaluated the effect of ISE on iNOS and COX-2 expression, which are involved in NO and PGE_2_ production, respectively. iNOS and COX-2 mRNA and protein levels were significantly increased by ISE treatment (Figures 2(c)–2(e)).

### 3.3. Prediction of Molecular Mechanisms of ISE Using Network Pharmacology Analysis

**Figure 3:**
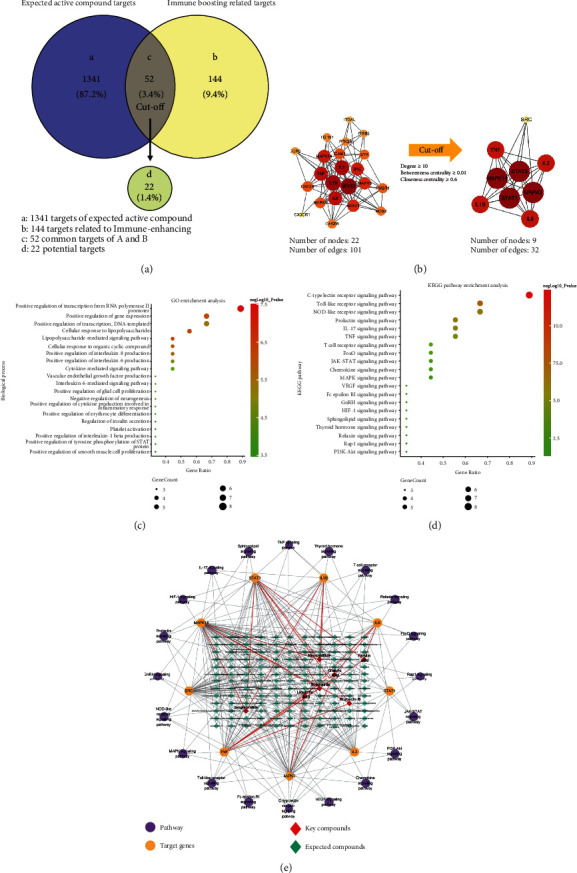
Network pharmacological analysis of ISE. (a) Venn diagram for 1393 target genes of ISE and 196 immune-enhancing related target genes. The 22 target genes were selected as potential targets of 52 common targets. (b) PPI networks of potential targets (left) and key targets (right). (c, d) Bubble chart of GO (c) and KEGG pathway (d) enrichment analysis for the 9 key targets. (e) C-T-P network.

Advances in network pharmacology analysis have provided a new approach for interpreting unclear molecular mechanisms of traditional medicine, including multiple components and their multiple-level actions [24]. To predict the key compounds and molecular mechanisms underlying the immune-enhancing effect, 2,419 chemical constituents of ten herbs in ISE were obtained from two herbal medicine databases: TM-MC and PubMed. Candidate active compounds were evaluated for their OB using the QED method. Among them, 1,602 chemicals had a QED value of 0.400 or higher and an OB value of TRUE. After excluding duplicate compounds, 143 chemical compounds were selected as potential active compounds of ISE (Supplementary Table S1). Target prediction databases, SwissTargetPrediction and GeneCards, were used to obtain 1,393 targets of the expected active compounds and 196 immune-enhancing-associated targets. Among the 52 common target genes, 22 that satisfied the cut-off values (relevance score ≥ 9) were selected as potential targets (Figure 3(a)). A protein-protein interaction (PPI) network was constructed and analyzed to obtain key targets from potential targets (Figure 3(b)). Among the 22 potential targets, *MAPK3*, *MAPK14*, *STAT1*, *STAT3*, *IL1B*, *IL2*, *IL6*, *TNF*, and *SRC* were selected as key targets that satisfied the criteria of the three indicators (degree, betweenness centrality, and closeness centrality) (Table 3). Specifically, *IL6* and *TNF* showed high degree values and high relevance scores. KEGG and GO pathway enrichment analyses of the nine key target genes showed that pathways associated with immune responses were enriched, such as the transcription from the RNA polymerase II promoter (GO:0045944) was positively regulated (Figure 3(c)). The KEGG pathway enrichment analysis also suggested that the C-type lectin receptor (CLR) signaling pathway was a key-enriched target (Figure 3(d)).

A compound-target-pathway (C-T-P) network was established to provide a comprehensive understanding of the network pharmacology (Figure 3(e)). The C-T-P network was composed of 172 nodes (143 predicted active compounds, nine key targets, and 20 pathways) and 298 edges. The seven red diamond nodes (scopoletin, neocnidilide, liquiritic acid, isoglabrolide, glabric acid, ferulic acid, and erybacin B) represent the highest degree values of other expected active compounds, and the red edge represents their interaction with key target genes, thereby indicating that they are important nodes and edges in the network. Taken together, it can be deduced that ISE modulates immune responses through the actions of its key compounds on TNF-*α*- and IL-6-involved biological pathways.

### 3.4. HPLC-QTOF-MS Analysis of ISE

**Figure 4:**
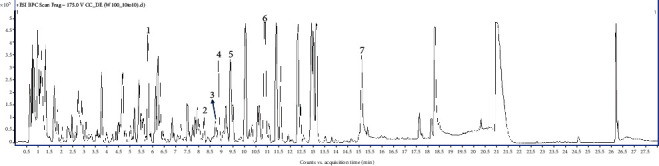
HPLC-QTOF-MS chromatogram of ISE. Representative base peak chromatogram of ISE in the positive ESI mode. Peaks of key compounds identified using forward scoring are tagged 1–7. 1, ferulic acid; 2, isoglabrolide; 3, neocnidilide; 4, glabric acid; 5, liquiritic acid; 6, scopoletin; and 7, erybacin B.

The optimal HPLC-QTOF-MS conditions were used to analyze ISE (Figure 4). Using the “Find by Auto MS/MS” function, seven key components (scopoletin, neocnidilide, liquiritic acid, isoglabrolide, glabric acid, ferulic acid, and erybacin B) were identified. Chemical information and content, such as compound name, molecular formula, retention time, adduct, calculated molecular ions, measured molecular ions (m/z), error (ppm), score, and origin quantified by LC-MS analysis are listed in Table 4.

### 3.5. ISE Enhances TNF-*α* and IL-6 Expression

**Figure 5:**
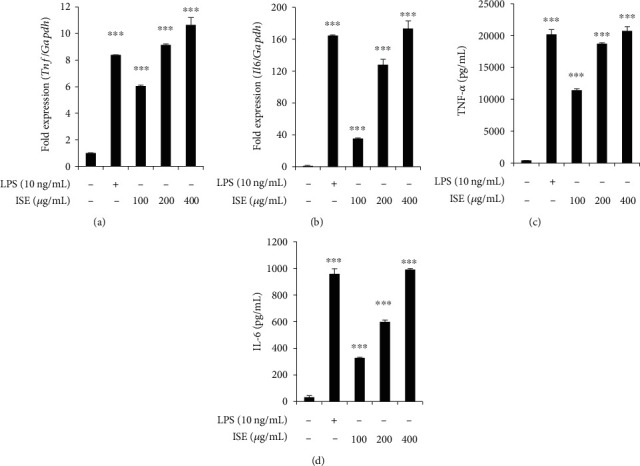
Effects of ISE on TNF-*α* and IL-6 mRNA and protein expression in RAW264.7 cells. (a, b) mRNA expression of TNF-*α* and IL-6 was analyzed using qRT-PCR. GAPDH was used as an internal control. (c, d) TNF-*α* secretion and IL-6 secretion were analyzed using ELISA. The data are shown as mean ± SEM of triplicate experiments.

The network pharmacology analysis revealed key target genes associated with the expected active compound targets and immune boosting-related genes. Among them, *TNF* and *IL6* were highly enriched in ISE (Table 2). TNF-*α* and IL-6 mRNA and protein levels were considerably increased by 100–400 *μ*g/mL of ISE in RAW264.7 cells (Figures 5(a)–5(d)). Considering these results collectively, we suggest that ISE exerts immune-enhancing effects by increasing *Tnf* and *Il6* expression in RAW264.7 cells.

### 3.6. ISE Is Associated with NF-*κ*B (p65) Activation

**Figure 6:**
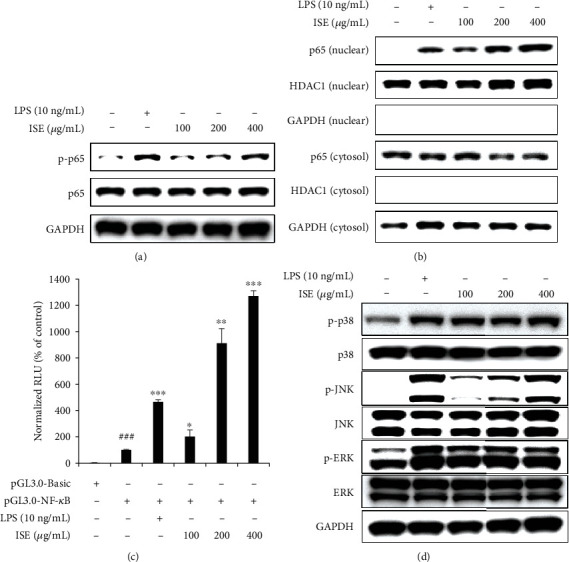
Effects of ISE on the activation of NF-*κ*B p65 and MAPK in RAW264.7 cells. (a, b) Protein expression of p-p65 and p65 was analyzed through western blotting. HDAC1 and GAPDH was used as an internal control. (c) Results of the NF-*κ*B reporter assay of ISE- and LPS-treated cells grown in 96-well plates, which were cotransfected with a pNF-*κ*B-Luc vector and a pNL-Luc vector using the FuGENE 6 Transfection Reagent. After cotransfection for 24 h, cells were treated with growth medium containing the indicated concentration of ISE or 10 ng/mL of LPS for 6 h. Following LPS or ISE treatment, the cells were lysed and luciferase activity was assessed using a dual-luciferase reporter assay system and a luminometer. (d) Protein expression of p-ERK1/2, ERK1/2, p-p38, p38, p-JNK, and JNK was analyzed through western blotting. GAPDH was used as an internal control. Data are shown as mean ± SEM of triplicate experiments.

Previous studies have demonstrated that NF-*κ*B is an essential transcriptional regulator of proinflammatory mediators and cytokines including iNOS, TNF-*α*, and IL-6 [25, 26]. Recent studies have shown that CLR-mediated signaling cascades activate the NF-*κ*B family and contribute to innate-immune and inflammatory responses [27]. The KEGG pathway enrichment analysis and C-T-P network revealed that the key targets were also enriched in the CLR signaling pathway (Figures 3(d) and 3(e)); therefore, ISE may modulate immune-enhancing effects through TNF-*α*- and IL-6-associated activation of NF-*κ*B (p65) and MAPK. To identify the mechanisms underlying the enhancement of *Tnf* and *Il6* expression by ISE, the phosphorylation status of p65 (a subunit of NF-*κ*B) was evaluated. ISE-treated cells exhibited enhanced p65 phosphorylation compared with that in LPS-treated cells (Figure 6(a)). The phosphorylation of p65 induces its translocation from the cytosol to the nucleus to enhance the expression of *Tnf* and *Il6* [28, 29]. The association between ISE treatment and p65 translocation was evaluated. ISE-treated cells showed a significant increase in the nuclear translocation of p65 compared with that in LPS-treated cells (Figure 6(b)). Furthermore, the NF-*κ*B reporter assay revealed that ISE-treated cells have markedly increased NF-*κ*B transactivation compared with that in LPS-treated cells (Figure 6(c)). Moreover, we evaluated the phosphorylation level of ERK1/2, p38, and JNK in RAW264.7 cells. ISE-treated cells exhibited elevation of ERK1/2, p38, and JNK phosphorylation compared with that in LPS-treated cells (Figure 6(d)). In ISE-treated cells, the phosphorylation level of JNK was strongly elevated in a concentration-dependent manner, whereas that of ERK and p38 was elevated in a concentration-independent manner.

### 3.7. Rapamycin Suppresses ISE-Induced NF-*κ*B Transactivation and mRNA Expression of Cytokines

**Figure 7:**
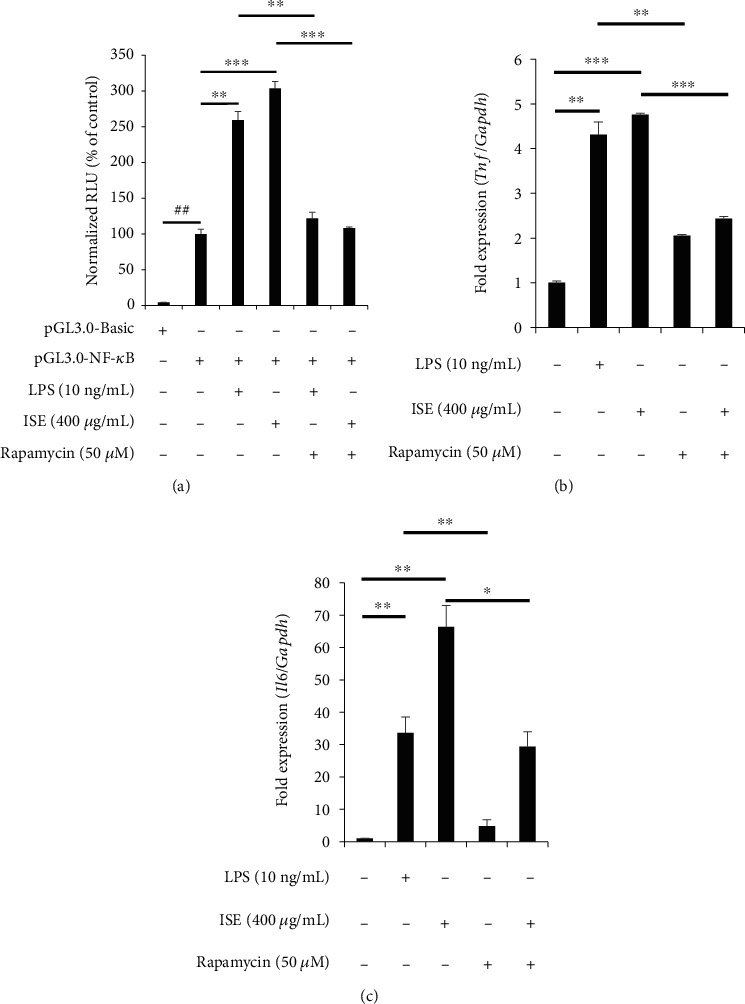
Effects of rapamycin on ISE-induced NF-*κ*B transactivation and mRNA expression of cytokines in RAW264.7 cells. (a) RAW264.7 cells grown in 96-well plates were cotransfected with a pNF-*κ*B-Luc vector and a pNL-Luc vector using FuGENE 6 Transfection Reagent. After cotransfection for 24 h, cells were treated with growth medium containing 400 *μ*g/mL of ISE or 10 ng/mL of LPS with absence or presence of 50 *μ*M of rapamycin for 6 h. Following LPS or ISE treatment, the cells were lysed and luciferase activity was assessed using a dual-luciferase reporter assay system and a luminometer. (b, c) mRNA expression of TNF-*α* and IL-6 was analyzed using qRT-PCR. GAPDH was used as an internal control. Data are shown as mean ± SEM of triplicate experiments.

Previous studies have shown that rapamycin inhibits NF-*κ*B activation and mRNA expression of proinflammatory cytokines, including TNF-*α* and IL-6 [30, 31]. The mechanism underlying the activation of NF-*κ*B by ISE treatment with or without rapamycin was evaluated. ISE with rapamycin-treated cells showed a reduction in NF-*κ*B transcriptional activity compared with that in ISE-treated cells (Figure 7(a)). Moreover, combined treatment with ISE and rapamycin decreased *Tnf* and *Il6* mRNA levels compared with that in cells treated with ISE alone (Figures 7(b) and 7(c)).

## 4. Discussion

ISE is frequently prescribed and widely used in many oriental medicines for the treatment of seasonal colds and Qi deficiency. ISE components, including *A. continentalis* Radix and *B. falcatum* Radix, have been shown to have pharmaceutical and health-promoting effects [32].

Several studies have reported that an immune approach is effective in controlling some infectious and chronic diseases [33–35]. Many traditional herbal medicines are clinically known to relieve symptoms of the aforementioned diseases; therefore, there have been attempts to identify more immunomodulatory herbal medicines [36]. However, few studies have elucidated the molecular mechanisms underlying the immunomodulatory activity of herbal medicines [37]. The chemical and pharmacological complexity of herbal medicines has made it difficult to study their mechanisms of action. However, the application of network pharmacology analysis has made it feasible to analyze the properties of herbal medicines as well as their molecular mechanisms. In this study, network interactions were established in conjunction with quantitative estimate of drug-likeness (QED), OB evaluation, PPI network analysis, and GO and KEGG enrichment analyses. The immune-enhancing effect of ISE was inferred to be associated with the seven key compounds identified, which were also determined using the HPLC-QTOF-MS analysis. Particularly for ferulic acid and scopoletin, these results are in line with previous reports showing their immune-enhancing effect [38, 39]. Besides, the nine key targets included TNF and IL-6 with the highest degree values and the C-type lectin receptor signaling pathway related to NF-*κ*B activation. Topological network analysis parameters can be also used to select key targets because they indicate the importance of nodes in the network. These selected key targets were suggested to be more relevant when compared to other targets. For example, COX-2 and iNOS were excluded from the list of key targets, although they were included in 1341 targets of expected active compounds, because they did not satisfy the topological cut-off values (degree ≥ 10, betweenness centrality ≥ 0.001, and closeness centrality ≥ 0.6). In other words, topological network analysis resulted in the identification of the nine key targets, especially TNF-*α* and IL-6, which are more relevant targets related to immune responses than the other targets. Furthermore, recent studies have shown that CLR-mediated signaling cascades activate the NF-*κ*B family [27]. Accordingly, ISE was deduced to modulate immune-enhancing effects through TNF-*α*- and IL-6-associated NF-*κ*B (p65) activation. Based on these outcomes, experimental validation was performed. In the current study, ISE was observed to exhibit immune-enhancing effects via the NF-*κ*B signaling pathway. The mode of action underlying the activity of ISE involved the translocation of p65 by increasing the phosphorylation of p65.

Macrophages are actively involved in immune responses by activating several signaling pathways that initiate the release of inflammatory mediators and proinflammatory cytokines, which assemble additional immune cells to the infected sites [38]. In the current study, ISE was found to be an effective enhancer of NO and PGE_2_ secretion that increased both the transcriptional and translational processes of iNOS and COX-2 in RAW264.7 cells. In addition, the mRNA expressions of *Tnf* and *Il6* were significantly increased by ISE. These results indicate that the enhancing effects of ISE contribute to the upregulation of the mRNA levels of these proinflammatory cytokines. Since the expression of proinflammatory mediators, including TNF-*α*, IL-6, iNOS, and COX-2, is regulated by NF-*κ*B binding to the *κ*B site [8], whether ISE promotes NF-*κ*B activation was determined by evaluating its phosphorylation, nuclear translocation, and transcriptional activity. The reporter assay indicated that NF-*κ*B transcriptional activity increased following ISE treatment in a dose-dependent way. Taken together, these data indicate that ISE promotes NF-*κ*B activation by increasing NF-*κ*B phosphorylation, thereby increasing NF-*κ*B nuclear translocation and resulting in the upregulation of iNOS, COX-2, TNF-*α*, and IL-6 protein levels. The inhibitor study demonstrated that rapamycin suppressed ISE-induced NF-*κ*B transcriptional activity and *Tnf* and *Il6* mRNA expression. Moreover, MAPK is known to control NF-*κ*B activation and regulate the production of proinflammatory cytokines [40–42]. These results showed that ISE provided immune-enhancing effect through the NF-*κ*B and MAPK signaling pathways in RAW264.7 cells. This molecular mechanism shows that ISE can be used as a pharmaceutical example for the development of immune response agonists through NF-*κ*B activation.

## 5. Conclusions

The findings of our study demonstrate that ISE significantly promotes immune regulators, including iNOS, COX-2, TNF-*α*, and IL-6, by activating the NF-*κ*B signaling pathway through phosphorylation, nuclear translocation, and transcriptional activity of NF-*κ*B in murine macrophages. The mechanism of action of ISE was predicted using network pharmacology analysis and validated with transcriptional and translational studies, including an NF-*κ*B inhibitor study. ISE is an efficient pharmaceutical agent for targeting the NF-*κ*B signaling pathway.

## Figures and Tables

**Table 1:** Composition of Insampaedok-san (IS).

No.	Scientific name	Origin	Dose (g)
1	*Glycyrrhiza uralensis* Rhizoma	China	1.25
2	*Ostericum koreanum* Radix	South Korea	1.25
3	*Platycodon grandiflorum* Radix	South Korea	1.25
4	*Aralia continentalis* Radix	South Korea	1.25
5	*Bupleurum falcatum* Radix	South Korea	1.25
6	*Panax ginseng* Radix	South Korea	1.25
7	*Angelica decursiva* Radix	South Korea	1.25
8	*Poria cocos* Sclerotium	North Korea	1.25
9	*Citrus aurantium* Fructus	China	1.25
10	*Cnidium officinale* Rhizoma	South Korea	1.25
	Total		12.5

**Table 2:** TaqMan® probes used in qRT-PCR amplifications.

Gene name	Assay ID	Assay information
*Inos*	Mm00440502_m1	TaqMan® Gene Expression Assay
*Cox2*	Mm00478374_m1	
*Tnf*	Mm00443258_m1	
*Il6*	Mm00446190_m1	
*Gapdh*	Mm99999915_g1	

**Table 3:** Key targets of ISE based on the results of PPI network topological analysis.

No.	UniProt ID	Gene	Degree	Relevance score
1	P27361	*MAPK3*	8	9.86
2	P40763	*STAT3*	8	9.45
3	Q16539	*MAPK14*	8	9.49
4	P42224	*STAT1*	8	10.01
5	P01584	*IL1B*	7	19.65
6	P05231	*IL6*	7	21.34
7	P01375	*TNF*	7	29.00
8	P60568	*IL2*	7	13.50
9	P12931	*SRC*	4	10.84

**Table 4:** Chemical information and quantitative analysis of authentic key components in ISE.

Peak no.	Compound name	Molecular formula	Degree in network	Correlating targets	Rt (min)	Adduct	Calculated molecular ions	Measured molecular ions (m/z)	Error (ppm)	Score	Origin
1	Ferulic acid	C_10_H_10_O_4_	3	*TNF* *IL6* *STAT3*	4.662	(M+H)^+^ [-H_2_O]	194.0573	177.0541	3.31	95.76	*O. koreanum*, *A. continentalis*, *P. ginseng*, *C. aurantium*, *C. officinale*
2	Isoglabrolide	C_30_H_44_O_4_	3	*IL1B* *MAPK14* *STAT3*	8.247	(M+H)^+^	468.324	469.3317	-0.83	99.34	*G. uralensis*
3	Neocnidilide	C_12_H_18_O_2_	3	*IL1B* *MAPK14* *STAT3*	8.748	(M+H)^+^	194.1307	195.1372	4.84	91.15	*C. officinale*
4	Glabric acid	C_30_H_46_O_5_	3	*TNF* *IL6* *MAPK3*	8.875	(M+H)^+^	486.334	487.3424	1.22	99.05	*G. uralensis*
5	Liquiritic acid	C_30_H_46_O_4_	3	*TNF* *IL6* *MAPK3*	9.404	(M+H)^+^ [-H_2_O]	470.3396	453.3367	-1.12	96.15	*G. uralensis*
6	Scopoletin	C_10_H_8_O_4_	4	*TNF* *IL1B* *MAPK14* *STAT3*	10.887	(M+H)^+^ [-H_2_O]	192.0414	175.0381	4.49	92.45	*O. koreanum*, *A. continentalis*, *A. decursiva*, *C. aurantium*, *C. officinale*
7	Erybacin B	C_19_H_18_O_5_	3	*TNF* *STAT3* *MAPK14*	15.375	(M+H)^+^	326.1154	327.1236	-2.09	96.77	*G. uralensis*

## Data Availability

The data presented in this study are available upon request from the corresponding authors.

## Supplementary Materials

Supplementary Materials The supplementary table is described in Supplementary Materials. Supplementary Table S1: list of physicochemical properties, QED, and OB of the expected active compounds from ISE.
