# Supplementary material for: Insampaedok-San Extract Exerts an Immune-Enhancing Effect through NF-κB p65 Pathway Activation
Source: Biomed Res Int. 2023 Sep 22;2023:5458504. doi: 10.1155/2023/5458504 (PMC10541303; doi:10.1155/2023/5458504)
Supplement: Supplementary Materials — The supplementary table is described in Supplementary Materials. Supplementary Table S1: list of physicochemical properties, QED, and OB of the expected active compounds from ISE. [file 5458504.f1.docx]

**Supplementary Materials for:**

**Insampaedok-san extract exerts an immune-enhancing effect through NF-κB p65 pathway activation**

Gyuwon Huh^1,2,†^, Youngse Oh^3,†^, Youngsic Jeon^1^, Ki Sung Kang^4^, Su Nam Kim^1^, Sang Hoon Jung^1,2^, Seung Hyun Kim^3,*^, and Young-Joo Kim^1,*^

**Affiliations**

*^1^Natural Product Research Center, Korea Institute of Science and Technology, Gangneung, Gangwon-do 25451, Korea*

*^2^Division of Bio-Medical Science & Technology, University of Science and Technology, Daejeon 34113, Korea*

*^3^College of Pharmacy, Yonsei Institute of Pharmaceutical Science, Yonsei University, Incheon, 21983, Korea*

*^4^College of Korean Medicine, Gachon University, Seongnam 13120, Republic of Korea*

*Correspondence should be addressed to Seung Hyun Kim; kimsh11@yonsei.ac.kr and Young-Joo Kim; yjkim7801@kist.re.kr

†Gyuwon Huh and Youngse Oh contributed equally to this work.

List of Contents

| no. | Content | Page |
| --- | --- | --- |
| 1 | **List of physicochemical properties, QED, and OB of expected active compounds from ISE** | S3~S8 |

**Table S1. List of physicochemical properties, QED, and OB of expected active compounds from ISE**

| **No.** | **Compound name** | **MW** | **ALOGP** | **HBA** | **HBD** | **PSA** | **ROTB** | **AROM** | **ALERTS** | **QED** | **OB** |
| --- | --- | --- | --- | --- | --- | --- | --- | --- | --- | --- | --- |
| 1 | 1-cyclohexene-1-carboxaldehyde | 110.16 | 1.68 | 1 | 0 | 17.07 | 1 | 0 | 1 | 0.469 | TRUE |
| 2 | 1-cyclohexene-1-carboxaldehyde, 4-(1-methylethyl)- | 152.24 | 2.47 | 1 | 0 | 17.07 | 2 | 0 | 1 | 0.555 | TRUE |
| 3 | 1R,6R-1-(3-heptyloxiran-2-yl)octa-7-ene-2,4-diyne-1,6-diol | 276.38 | 3.13 | 3 | 2 | 52.99 | 8 | 0 | 3 | 0.407 | TRUE |
| 4 | 2Z,9Z-heptadecadiene-4,6-diyne-1-ol | 244.38 | 1.01 | 1 | 1 | 20.23 | 8 | 0 | 2 | 0.499 | TRUE |
| 5 | 3,4,5-trimethoxy-trans-cinnamic acid | 238.24 | 1.72 | 5 | 1 | 64.99 | 5 | 1 | 1 | 0.791 | TRUE |
| 6 | 3R,4E,8S,9Z-heptadeca-1,4,9-triene-6-yne-3,8-diol | 262.39 | 3.99 | 2 | 2 | 40.46 | 9 | 0 | 2 | 0.487 | TRUE |
| 7 | 4-methyl-3-trans-hexenylferulate | 290.36 | 3.58 | 4 | 1 | 55.76 | 8 | 1 | 2 | 0.587 | TRUE |
| 8 | 6,7-dimethoxy-coumarin | 206.20 | 1.84 | 4 | 0 | 48.67 | 2 | 2 | 1 | 0.703 | TRUE |
| 9 | 9-epoxy-falcarindiol | 276.38 | 3.13 | 3 | 2 | 52.99 | 8 | 0 | 3 | 0.407 | TRUE |
| 10 | angenomalin | 352.39 | 4.05 | 5 | 0 | 65.11 | 6 | 2 | 3 | 0.446 | TRUE |
| 11 | bergaptol | 202.17 | 1.77 | 4 | 1 | 63.58 | 0 | 3 | 1 | 0.564 | TRUE |
| 12 | bicyclo[3.1.1]hept-2-ene-2-carboxaldehyde, 6,6-dimethyl- | 150.22 | 2.38 | 1 | 0 | 17.07 | 1 | 0 | 1 | 0.524 | TRUE |
| 13 | crithmumdiol | 262.39 | 3.98 | 2 | 2 | 40.46 | 9 | 0 | 2 | 0.487 | TRUE |
| 14 | esculetin | 178.14 | 1.12 | 4 | 2 | 70.67 | 0 | 2 | 2 | 0.468 | TRUE |
| 15 | falcarindiol | 260.38 | 3.75 | 2 | 2 | 40.46 | 8 | 0 | 2 | 0.515 | TRUE |
| 16 | ferulic acid | 194.19 | 1.36 | 4 | 2 | 66.76 | 3 | 1 | 1 | 0.713 | TRUE |
| 17 | imperatorin | 270.28 | 3.31 | 4 | 0 | 52.58 | 3 | 3 | 2 | 0.541 | TRUE |
| 18 | isopropylferulate | 236.27 | 2.41 | 4 | 1 | 55.76 | 5 | 1 | 1 | 0.798 | TRUE |
| 19 | nodakenetin | 246.26 | 2.09 | 4 | 1 | 59.67 | 1 | 2 | 1 | 0.783 | TRUE |
| 20 | notoptol | 354.40 | 3.86 | 5 | 1 | 72.81 | 6 | 3 | 2 | 0.537 | TRUE |
| 21 | ostruthin | 298.38 | 4.46 | 3 | 1 | 50.44 | 5 | 2 | 2 | 0.655 | TRUE |
| 22 | phellopterin | 300.31 | 3.36 | 5 | 0 | 61.81 | 4 | 3 | 2 | 0.546 | TRUE |
| 23 | pregnenolone | 316.49 | 3.97 | 2 | 1 | 37.3 | 1 | 0 | 1 | 0.743 | TRUE |
| 24 | scopoletin | 192.17 | 1.52 | 4 | 1 | 59.67 | 1 | 2 | 1 | 0.696 | TRUE |
| 25 | trans-ferulic acid | 194.19 | 1.36 | 4 | 2 | 66.76 | 3 | 1 | 1 | 0.713 | TRUE |
| 26 | 5,6,7,4'-tetramethoxyflavone | 342.34 | 3.01 | 6 | 0 | 67.13 | 5 | 3 | 0 | 0.710 | TRUE |
| 27 | 6-hydroxymelatonin | 248.28 | 1.51 | 3 | 3 | 74.35 | 5 | 2 | 0 | 0.750 | TRUE |
| 28 | arnebifuranone | 316.35 | 2.76 | 5 | 0 | 65.74 | 7 | 1 | 2 | 0.571 | TRUE |
| 29 | caffeic acid | 180.16 | 0.93 | 4 | 3 | 77.76 | 2 | 1 | 2 | 0.467 | TRUE |
| 30 | α-spinasterol glucoside | 146.18 | 1.03 | 3 | 1 | 46.53 | 5 | 0 | 0 | 0.630 | TRUE |
| 31 | (2R)-2-(acetoxymethyl)-1,3,5-trimethyl-4-(3-methyl-2-buten-1-yl)cyclohexan-1-ol | 282.42 | 3.44 | 3 | 1 | 46.53 | 5 | 0 | 1 | 0.786 | TRUE |
| 32 | (3S,3aR)-3-butyl-3a,4,5,6-tetrahydro-3H-2-benzofuran-1-one | 194.27 | 2.86 | 2 | 0 | 26.3 | 3 | 0 | 0 | 0.690 | TRUE |
| 33 | 1,8-dimethyl-4-(1-methylethyl)-spiro[4.5]dec-8-en-7-one | 220.36 | 3.71 | 1 | 0 | 17.07 | 1 | 0 | 0 | 0.658 | TRUE |
| 34 | 1-[3-(benzyloxy)-5-hydroxybenzyl]-1,2,3,4-tetrahydro-6-methoxyisoquinoline | 375.47 | 3.94 | 4 | 2 | 50.72 | 6 | 3 | 0 | 0.685 | TRUE |
| 35 | 2,3-dimethyl-3-decanol | 186.34 | 3.69 | 1 | 1 | 20.23 | 7 | 0 | 0 | 0.643 | TRUE |
| 36 | 2-pinen-4-one | 150.22 | 2.25 | 1 | 0 | 17.07 | 0 | 0 | 0 | 0.517 | TRUE |
| 37 | 4-(1-methylethyl)-2-cyclohexen-1-one | 138.21 | 2.06 | 1 | 0 | 17.07 | 1 | 0 | 0 | 0.542 | TRUE |
| 38 | columbianadin | 328.36 | 3.46 | 5 | 0 | 65.74 | 4 | 2 | 2 | 0.640 | TRUE |
| 39 | continentalic acid | 302.46 | 4.56 | 2 | 1 | 37.3 | 2 | 0 | 1 | 0.759 | TRUE |
| 40 | ent-continentalic acid | 302.46 | 4.56 | 2 | 1 | 37.3 | 2 | 0 | 1 | 0.759 | TRUE |
| 41 | eugenol | 164.20 | 2.25 | 2 | 1 | 29.46 | 3 | 1 | 1 | 0.694 | TRUE |
| 42 | 4'-senecioylkhellactone | 342.40 | 3.26 | 5 | 1 | 68.9 | 3 | 2 | 3 | 0.531 | TRUE |
| 43 | D-laserpitin | 344.36 | 2.55 | 6 | 1 | 85.97 | 3 | 2 | 2 | 0.686 | TRUE |
| 44 | adenosine | 267.25 | -1.50 | 7 | 4 | 139.54 | 2 | 2 | 0 | 0.522 | TRUE |
| 45 | deltoin | 328.36 | 3.46 | 5 | 0 | 65.74 | 4 | 2 | 2 | 0.640 | TRUE |
| 46 | laserpitin | 450.57 | 1.30 | 7 | 2 | 110.13 | 7 | 0 | 1 | 0.563 | TRUE |
| 47 | peujaponisinol B | 344.36 | 2.55 | 6 | 1 | 85.97 | 3 | 2 | 2 | 0.686 | TRUE |
| 48 | qianhucoumarin A | 344.36 | 2.64 | 6 | 1 | 85.97 | 3 | 2 | 2 | 0.686 | TRUE |
| 49 | apigenin | 270.24 | 2.11 | 5 | 3 | 90.9 | 1 | 3 | 0 | 0.678 | TRUE |
| 50 | hesperetin | 302.28 | 1.91 | 6 | 3 | 96.22 | 2 | 2 | 0 | 0.784 | TRUE |
| 51 | hesperitin | 302.28 | 1.91 | 6 | 3 | 96.22 | 2 | 2 | 0 | 0.784 | TRUE |
| 52 | kaempferol | 286.24 | 1.58 | 6 | 4 | 111.13 | 1 | 3 | 0 | 0.582 | TRUE |
| 53 | naringenin | 272.26 | 1.84 | 5 | 3 | 86.99 | 1 | 2 | 0 | 0.737 | TRUE |
| 54 | obacunoic acid | 472.53 | 2.57 | 8 | 2 | 126.57 | 4 | 1 | 2 | 0.501 | TRUE |
| 55 | p-menth-1-en-9-al | 152.24 | 2.35 | 1 | 0 | 17.07 | 2 | 0 | 2 | 0.438 | TRUE |
| 56 | quercetin | 302.24 | 1.23 | 7 | 5 | 131.36 | 1 | 3 | 1 | 0.460 | TRUE |
| 57 | scoparone | 206.20 | 1.84 | 4 | 0 | 48.67 | 2 | 2 | 1 | 0.709 | TRUE |
| 58 | tetramethoxyflavone | 342.30 | 3.03 | 6 | 0 | 67.13 | 5 | 3 | 0 | 0.764 | TRUE |
| 59 | (Z)-6,8,7,3-diligustilide | 380.48 | 4.52 | 4 | 0 | 52.6 | 4 | 0 | 1 | 0.673 | TRUE |
| 60 | 1,4,5,6,7,7a-hexahydro-4-methyl-7-2H-inden-2-one | 192.30 | 2.98 | 1 | 0 | 17.07 | 1 | 0 | 0 | 0.624 | TRUE |
| 61 | 1-phenyl-1-pentanone | 162.23 | 2.93 | 1 | 0 | 17.07 | 4 | 1 | 0 | 0.665 | TRUE |
| 62 | 2-methoxy-4-vinylphenol | 150.18 | 2.14 | 2 | 1 | 29.46 | 2 | 1 | 0 | 0.699 | TRUE |
| 63 | 3,8-dihydro-diligustilide | 382.50 | 4.73 | 4 | 0 | 52.6 | 5 | 0 | 1 | 0.645 | TRUE |
| 64 | 3R-butyl-4,5-dihydrophthalide | 192.26 | 2.71 | 2 | 0 | 26.3 | 3 | 0 | 0 | 0.687 | TRUE |
| 65 | 3β,7β-dihydroxy-11,15,23-trioxo-lanost-8,16-dien-26-oic acid | 514.66 | 3.17 | 7 | 3 | 128.97 | 6 | 0 | 0 | 0.496 | TRUE |
| 66 | 4,5-dihydro-3-butylphthalide | 192.26 | 2.71 | 2 | 0 | 26.3 | 3 | 0 | 0 | 0.687 | TRUE |
| 67 | Z-6,7-epoxyligustilide | 206.24 | 2.15 | 3 | 0 | 38.83 | 2 | 0 | 1 | 0.650 | TRUE |
| 68 | Z-6,8',7,3'-diligustilide | 380.50 | 4.51 | 4 | 0 | 52.6 | 4 | 0 | 1 | 0.674 | TRUE |
| 69 | Z-ligustilide dimer E-232 | 382.50 | 4.54 | 4 | 0 | 52.6 | 4 | 0 | 1 | 0.670 | TRUE |
| 70 | angelicide | 380.48 | 4.51 | 4 | 0 | 52.6 | 4 | 0 | 1 | 0.674 | TRUE |
| 71 | cnidilide | 194.27 | 2.72 | 2 | 0 | 26.3 | 3 | 0 | 1 | 0.646 | TRUE |
| 72 | coniferyl ferulate | 356.37 | 3.25 | 6 | 2 | 85.22 | 8 | 2 | 1 | 0.706 | TRUE |
| 73 | neocnidilide | 194.27 | 2.86 | 2 | 0 | 26.3 | 3 | 0 | 0 | 0.690 | TRUE |
| 74 | octyl acetate | 172.27 | 2.90 | 2 | 0 | 26.3 | 8 | 0 | 0 | 0.562 | TRUE |
| 75 | riligustilide | 380.48 | 4.52 | 4 | 0 | 52.6 | 4 | 0 | 1 | 0.673 | TRUE |
| 76 | sedanenolide | 192.26 | 2.71 | 2 | 0 | 26.3 | 3 | 0 | 0 | 0.687 | TRUE |
| 77 | sedanonic acid lactone | 192.26 | 2.96 | 2 | 0 | 26.3 | 2 | 0 | 0 | 0.672 | TRUE |
| 78 | senkyunolide | 192.26 | 2.71 | 2 | 0 | 26.3 | 3 | 0 | 0 | 0.687 | TRUE |
| 79 | senkyunolide A | 192.26 | 2.71 | 2 | 0 | 26.3 | 3 | 0 | 0 | 0.687 | TRUE |
| 80 | senkyunolide M | 278.35 | 2.59 | 4 | 1 | 63.6 | 5 | 0 | 0 | 0.839 | TRUE |
| 81 | senkyunolide P | 382.50 | 4.73 | 4 | 0 | 52.6 | 5 | 0 | 1 | 0.645 | TRUE |
| 82 | senkyunolide Q | 264.37 | 3.31 | 3 | 1 | 46.53 | 5 | 0 | 0 | 0.828 | TRUE |
| 83 | tokinolide B | 380.48 | 4.56 | 4 | 0 | 52.6 | 4 | 0 | 1 | 0.671 | TRUE |
| 84 | valerophenone | 162.23 | 2.93 | 1 | 0 | 17.07 | 4 | 1 | 0 | 0.665 | TRUE |
| 85 | wallichilide | 412.53 | 4.42 | 5 | 0 | 69.67 | 8 | 0 | 1 | 0.552 | TRUE |
| 86 | (2Z,8Z,10E)-heptadecatriene-4,6-diyne-1,14-diol | 258.36 | 3.44 | 2 | 2 | 40.46 | 10 | 0 | 2 | 0.465 | TRUE |
| 87 | 1-(1-cyclohexen-1-yl)-ethanone | 124.18 | 2.07 | 1 | 0 | 17.07 | 1 | 0 | 0 | 0.523 | TRUE |
| 88 | 1,2-dimethoxy-4-(1-propenyl)-benzene | 178.23 | 2.78 | 2 | 0 | 18.46 | 3 | 1 | 0 | 0.708 | TRUE |
| 89 | 4-dimethyl-3-cyclohexene-1-acetaldehyde | 152.24 | 2.35 | 1 | 0 | 17.07 | 2 | 0 | 2 | 0.438 | TRUE |
| 90 | nerolidol | 222.37 | 4.30 | 1 | 1 | 20.23 | 7 | 0 | 1 | 0.636 | TRUE |
| 91 | 1,8-heptadecadiene-4,6-diyne-3,10-diol | 260.38 | 3.79 | 2 | 2 | 40.46 | 8 | 0 | 2 | 0.515 | TRUE |
| 92 | 2,4a-methanonaphthalen-7(4aH)-one, 1,2,3,4,5,6-hexa-hydro-1,1,5,5-tetramethyl-, (2s-cis)- | 218.34 | 3.52 | 1 | 0 | 17.07 | 0 | 0 | 0 | 0.608 | TRUE |
| 93 | 3-acetyl-1-(3,4-dimethoxyphenyl)-5-ethyl-4,5-dihydro-7,8-dimethoxy-4-methylene-3H-2,3-benzodiazepine | 424.50 | 3.75 | 6 | 0 | 69.59 | 7 | 2 | 0 | 0.674 | TRUE |
| 94 | 3-ethyl-2-hydroxy-2-cyclopenten-1-one | 126.16 | 1.16 | 2 | 1 | 37.3 | 1 | 0 | 0 | 0.572 | TRUE |
| 95 | ginsenoyne A | 258.36 | 3.74 | 2 | 1 | 32.76 | 8 | 0 | 3 | 0.408 | TRUE |
| 96 | oleanolic acid | 456.71 | 6.07 | 3 | 2 | 57.53 | 1 | 0 | 1 | 0.471 | TRUE |
| 97 | panaxydiol | 260.38 | 3.79 | 2 | 2 | 40.46 | 8 | 0 | 2 | 0.515 | TRUE |
| 98 | Glisoflavone | 368.40 | 3.34 | 6 | 3 | 100.13 | 4 | 3 | 2 | 0.519 | TRUE |
| 99 | Licocoumarin A | 406.50 | 5.07 | 5 | 3 | 90.9 | 5 | 3 | 2 | 0.438 | TRUE |
| 100 | 11-deoxoglycyrrhetinic | 456.71 | 6.04 | 3 | 2 | 57.53 | 1 | 0 | 1 | 0.473 | TRUE |
| 101 | 2'-hydroxyisolupalbigenin | 422.50 | 4.59 | 6 | 4 | 111.13 | 5 | 3 | 1 | 0.482 | TRUE |
| 102 | 3,4-didehydroglabridin | 322.40 | 3.38 | 4 | 2 | 58.92 | 1 | 2 | 1 | 0.790 | TRUE |
| 103 | 7,4'-Dihydroxyflavone | 254.24 | 2.40 | 4 | 2 | 70.67 | 1 | 3 | 0 | 0.753 | TRUE |
| 104 | 6,8-Diprenylgenistein | 406.50 | 4.95 | 5 | 3 | 90.9 | 5 | 3 | 1 | 0.562 | TRUE |
| 105 | 7-O-Methylluteone | 368.40 | 3.48 | 6 | 3 | 100.13 | 4 | 3 | 1 | 0.656 | TRUE |
| 106 | abyssinone II | 324.40 | 3.53 | 4 | 2 | 66.76 | 3 | 2 | 1 | 0.848 | TRUE |
| 107 | calycosin | 284.26 | 2.30 | 5 | 2 | 79.9 | 2 | 3 | 0 | 0.812 | TRUE |
| 108 | daidzein | 254.24 | 2.24 | 4 | 2 | 70.67 | 1 | 3 | 0 | 0.752 | TRUE |
| 109 | Erybacin B | 326.30 | 2.86 | 5 | 3 | 86.99 | 3 | 2 | 0 | 0.807 | TRUE |
| 110 | Eurycarpin A | 338.40 | 3.34 | 5 | 3 | 90.9 | 3 | 3 | 1 | 0.686 | TRUE |
| 111 | Formononetin | 268.26 | 2.66 | 4 | 1 | 59.67 | 2 | 3 | 0 | 0.835 | TRUE |
| 112 | Gancaonin D | 384.40 | 2.74 | 7 | 4 | 120.36 | 5 | 3 | 1 | 0.537 | TRUE |
| 113 | Gancaonin G | 352.40 | 3.87 | 5 | 2 | 79.9 | 4 | 3 | 1 | 0.750 | TRUE |
| 114 | Gancaonin M | 352.40 | 3.88 | 5 | 2 | 79.9 | 4 | 3 | 1 | 0.750 | TRUE |
| 115 | Gancaonin N | 368.40 | 3.53 | 6 | 3 | 100.13 | 4 | 3 | 1 | 0.656 | TRUE |
| 116 | Genistein | 270.24 | 2.04 | 5 | 3 | 90.9 | 1 | 3 | 0 | 0.677 | TRUE |
| 117 | Genkwanin | 284.26 | 2.50 | 5 | 2 | 79.9 | 2 | 3 | 0 | 0.813 | TRUE |
| 118 | Glabric acid | 486.70 | 4.42 | 5 | 3 | 94.83 | 1 | 0 | 0 | 0.510 | TRUE |
| 119 | Glabrolide | 468.70 | 5.2 | 4 | 1 | 63.6 | 0 | 0 | 0 | 0.531 | TRUE |
| 120 | Glicophenone | 358.40 | 2.98 | 6 | 4 | 107.22 | 6 | 2 | 1 | 0.592 | TRUE |
| 121 | Glicoricone | 368.40 | 3.31 | 6 | 3 | 100.13 | 4 | 3 | 1 | 0.658 | TRUE |
| 122 | Glyasperin F | 354.40 | 2.72 | 6 | 3 | 96.22 | 1 | 2 | 0 | 0.729 | TRUE |
| 123 | Glyasperin N | 420.50 | 4.34 | 6 | 3 | 100.13 | 3 | 3 | 1 | 0.587 | TRUE |
| 124 | Glycycoumarin | 368.40 | 3.53 | 6 | 3 | 100.13 | 4 | 3 | 2 | 0.518 | TRUE |
| 125 | Glycyrdione A | 408.50 | 4.75 | 5 | 3 | 94.83 | 8 | 2 | 2 | 0.433 | TRUE |
| 126 | Glycyrrhetol | 456.70 | 5.40 | 3 | 2 | 57.53 | 1 | 0 | 0 | 0.555 | TRUE |
| 127 | Glycyrrhisoflavanone | 368.40 | 2.92 | 6 | 2 | 85.22 | 2 | 2 | 0 | 0.848 | TRUE |
| 128 | Glyurallin A | 352.40 | 3.93 | 5 | 2 | 72.06 | 3 | 3 | 1 | 0.776 | TRUE |
| 129 | Glyuranolide | 512.70 | 4.56 | 6 | 1 | 89.9 | 2 | 0 | 1 | 0.546 | TRUE |
| 130 | Isoformononetin | 268.26 | 2.65 | 4 | 1 | 59.67 | 2 | 3 | 0 | 0.835 | TRUE |
| 131 | Isoglabrolide | 468.70 | 5.30 | 4 | 1 | 63.6 | 0 | 0 | 0 | 0.524 | TRUE |
| 132 | Kanzonol H | 424.50 | 5.01 | 5 | 2 | 68.15 | 4 | 2 | 1 | 0.676 | TRUE |
| 133 | Kanzonol S | 414.40 | 2.56 | 8 | 4 | 133.52 | 6 | 2 | 2 | 0.419 | TRUE |
| 134 | Licoagrochalcone D | 354.40 | 3.08 | 5 | 2 | 75.99 | 5 | 2 | 1 | 0.807 | TRUE |
| 135 | Licoflavanone | 340.40 | 3.33 | 5 | 3 | 86.99 | 3 | 2 | 1 | 0.746 | TRUE |
| 136 | Licofuranone | 356.40 | 2.89 | 6 | 3 | 96.22 | 4 | 2 | 1 | 0.729 | TRUE |
| 137 | Licoisoflavone A | 354.40 | 3.14 | 6 | 4 | 111.13 | 3 | 3 | 1 | 0.579 | TRUE |
| 138 | Licoricone | 382.40 | 3.66 | 6 | 2 | 89.13 | 5 | 3 | 1 | 0.705 | TRUE |
| 139 | Liquiritic acid | 470.70 | 5.17 | 4 | 2 | 74.6 | 1 | 0 | 0 | 0.554 | TRUE |
| 140 | Lupiwighteone | 338.40 | 3.51 | 5 | 3 | 90.9 | 3 | 3 | 1 | 0.684 | TRUE |
| 141 | Luteone | 354.40 | 3.11 | 6 | 4 | 111.13 | 3 | 3 | 1 | 0.579 | TRUE |
| 142 | Pratensein | 300.26 | 2.17 | 6 | 3 | 100.13 | 2 | 3 | 0 | 0.722 | TRUE |
| 143 | Wighteone | 338.40 | 3.51 | 5 | 3 | 90.9 | 3 | 3 | 1 | 0.684 | TRUE |
